# Assessment of platelet-to-white blood cell ratio on short-term mortality events in patients hospitalized with acute decompensated heart failure: evidence from a cohort study from Jiangxi, China

**DOI:** 10.3389/fcvm.2025.1454933

**Published:** 2025-02-07

**Authors:** Xin Huang, Maobin Kuang, Jiajun Qiu, Chao Wang, Guotai Sheng, Yang Zou, Guobo Xie

**Affiliations:** ^1^ Jiangxi Medical College, Nanchang University, Nanchang, Jiangxi, China; ^2^ Jiangxi Cardiovascular Research Institute, Jiangxi Provincial People’s Hospital, The First Affiliated Hospital of Nanchang Medical College, Nanchang, Jiangxi, China; ^3^ Jiangxi Provincial Geriatric Hospital, Jiangxi Provincial People’s Hospital, The First Affiliated Hospital of Nanchang Medical College, Nanchang, Jiangxi, China; ^4^ Department of Cardiology, Jiangxi Provincial People’s Hospital, The First Affiliated Hospital of Nanchang Medical College, Nanchang, Jiangxi, China

**Keywords:** platelet-to-white blood cell ratio, short-term prognostic, PWR, acute decompensated heart failure, ADHF

## Abstract

**Objective:**

Platelet-to-white blood cell ratio (PWR) as a comprehensive indicator of inflammatory response has been widely used to assess the prognosis of various diseases. However, the relationship between PWR and adverse outcomes in patients with acute decompensated heart failure (ADHF) remains unclear. This study aimed to evaluate the association between PWR and all-cause mortality within 30 days of hospitalization in ADHF patients from Jiangxi, China.

**Methods:**

A total of 1,453 ADHF patients from the Jiangxi-ADHF study1 cohort were included. The primary outcome measure was all-cause mortality within 30 days of hospitalization. Multivariable Cox proportional hazards regression, restricted cubic spline regression, and receiver operating characteristic curve analysis were employed to explore the association between the inflammatory marker PWR and all-cause mortality in ADHF patients within 30 days of hospitalization.

**Results:**

During the 30-day observation period, a total of 53 subjects experienced mortality events. Multivariable Cox regression showed a negative correlation between PWR and all-cause mortality within 30 days of hospitalization in ADHF patients. Restricted cubic spline regression demonstrated an L-shaped association between PWR and 30-day mortality risk (*p* for nonlinear = 0.038). Further threshold analysis revealed a threshold point for PWR at 15.88, where a decrease in PWR below this threshold was significantly associated with increased risk of all-cause mortality (*p* for log-likelihood ratio test = 0.046). Additionally, the results of receiver operating characteristic curve analysis indicated that PWR had high predictive accuracy for mortality events within 30 days of hospitalization in ADHF patients and is significantly better than the traditional HF marker N-Terminal Pro-Brain Natriuretic Peptide (AUC: NT-proBNP 0.69, PWR 0.76; Delong test *P* < 0.05). Subgroup analysis showed that compared to subjects with reduced or moderately reduced ejection fraction, ADHF patients with preserved ejection fraction had a lower risk of short-term mortality associated with PWR (HR:0.99 vs. 0.98 vs. 0.87, *P* for interaction = 0.0067).

**Conclusion:**

This study reveals, for the first time, a negative correlation between the inflammatory marker PWR and all-cause mortality within 30 days of hospitalization in ADHF patients. Based on the threshold analysis findings, patients with ADHF and a PWR below 15.88 had a significantly higher risk of death within 30 days.

## Introduction

Acute decompensated heart failure (ADHF) is a life-threatening emergency characterized by symptoms and signs of pulmonary and systemic congestion, such as exertional dyspnea, orthopnea, and bilateral lower extremity edema (1, 2), often requiring hospitalization to improve prognosis (3, 4). Although a significant proportion of patients are discharged from the hospital due to symptom improvement, many still experience in-hospital mortality or readmission within a short period (5–8). Epidemiological studies have shown that the mortality rate within 30 days of hospitalization for ADHF patients is approximately 10%, and the readmission rate within 30 days is around 25%, significantly impacting the lives of ADHF patients (9–11). With advancements in medical technology, numerous biomarkers have been discovered for risk assessment in ADHF patients (12). However, considering the complex pathophysiology of ADHF, single markers may not accurately reflect the severity of the disease (12). Studies have found that the deterioration of ADHF may be associated with neurohormonal activation, cell apoptosis, and inflammatory cascade reactions (13). These changes can cause endothelial cell damage and fluid homeostasis imbalance, exacerbating systemic organ load and leading to adverse outcomes (14, 15). Therefore, it is necessary to incorporate indicators measuring these mechanisms into the risk assessment of ADHF patients upon admission.

Platelet-to-white blood cell ratio (PWR) is a systemic inflammation indicator that has garnered attention in recent years, first proposed by Toutouzas et al. It is calculated as the platelet (PLT) count divided by the white blood cell (WBC) count and is primarily used to assess the degree of inflammation in the body (16). Subsequently, further studies have shown that this parameter plays a key role in risk assessment and prognosis across a wide range of diseases. Specifically, current evidence supports that PWR can be used to assess the risk of common chronic diseases such as diabetes, chronic kidney disease, cerebral white matter lesions, sarcopenia, and stroke (17–22). Additionally, PWR can be used for risk prediction and prognosis assessment of a variety of critical diseases (23–35), which has the potential for a wide range of disciplinary applications. It is also worth noting that evidence from the German cohort study (MyoVasc) suggests that PWR plays an important role in assessing adverse clinical outcomes in HF patients (36). Considering the serious adverse short-term prognosis of ADHF patients, further clarification of the role and value of PWR in risk assessment of short-term prognosis in ADHF patients is of great significance.

## Methods

### Study design and data source

**Figure 1:**
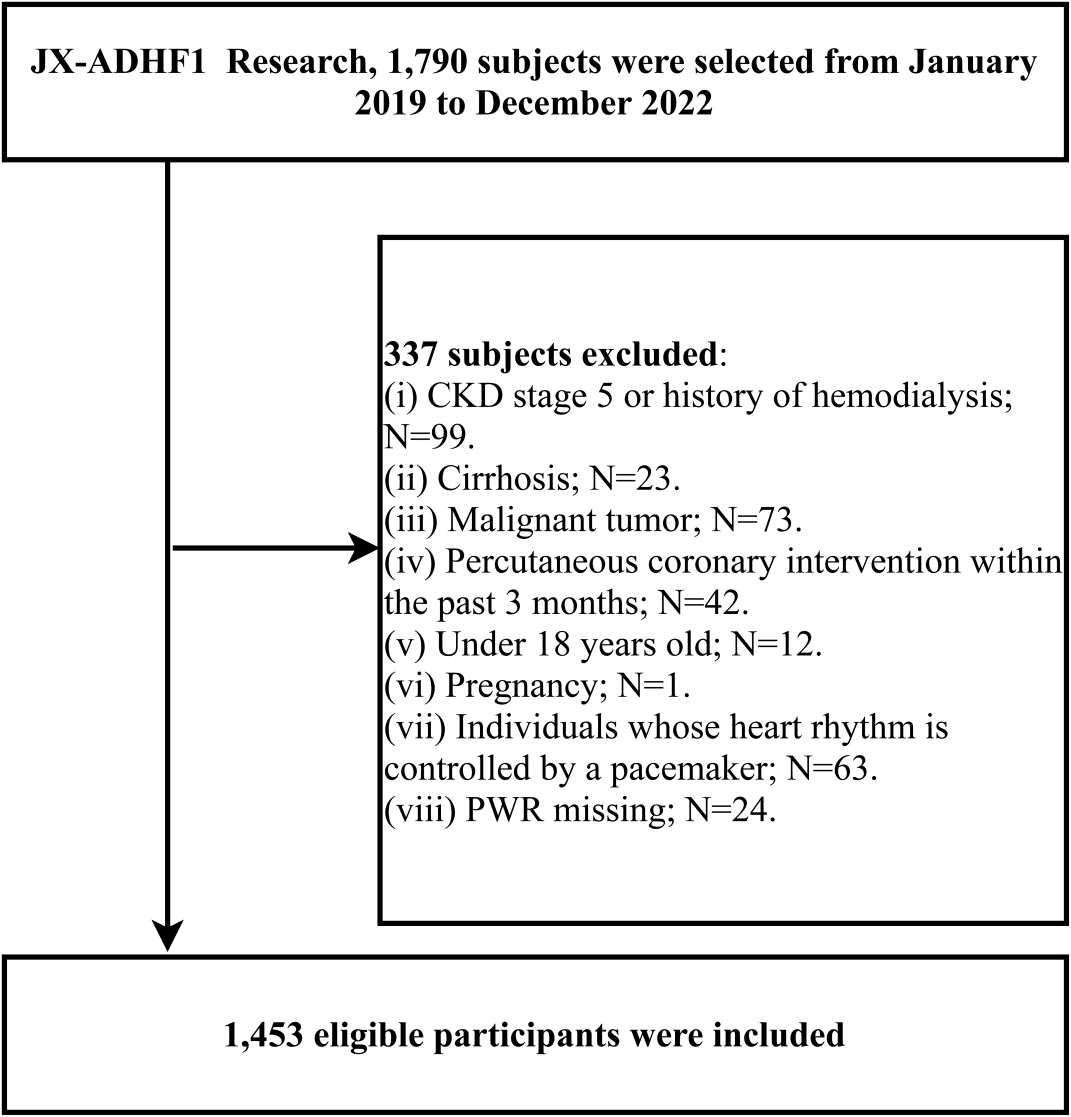
Flow chart for inclusion and exclusion of study participants.

This retrospective cohort study [Jiangxi-ADHF study1] included 1,790 ADHF patients who visited Jiangxi Provincial People's Hospital from January 2019 to December 2,022. The diagnosis of ADHF referred to the latest European Society of Cardiology (ESC) guidelines for the diagnosis and treatment of acute and chronic HF available in the year of hospitalization. Among the 1,790 patients, we excluded participants with the following characteristics: 23 participants with cirrhosis; 99 participants with stage 5 chronic kidney disease or a history of hemodialysis; 63 participants with pacemakers; 42 participants who underwent percutaneous coronary intervention within the last 3 months; 73 participants with malignancies; 1 participant with concomitant pregnancy; 12 participants under the age of 18; and 24 participants with missing PWR data. Ultimately, 1,453 subjects were included in this study. The detailed inclusion and exclusion process was shown in Figure 1.

### Ethical approval

The cohort study was conducted following the Helsinki Declaration. The use of research data was approved by the participants, and the study protocol was approved by the Ethics Committee of Jiangxi Provincial People's Hospital (IRB 2024−01).

### Measurement and assessment of baseline information

Baseline data were collected and recorded by professional medical staff upon the patient's admission, including demographic information (age, gender), lifestyle habits (smoking, drinking), blood pressure (BP), left ventricular ejection fraction (LVEF), New York Heart Association (NYHA) functional classification, clinical comorbidities [hypertension, diabetes mellitus, coronary heart disease (CHD), cerebral infarction], etc.

Blood specimens were obtained within 24 h of the patient's admission to the hospital and measured using automated analyzers, including N-Terminal Pro-Brain Natriuretic Peptide (NT-proBNP), WBC, red blood cell count, hemoglobin, PLT, albumin, alanine aminotransferase (ALT), aspartate aminotransferase (AST), creatinine, blood urea nitrogen, total cholesterol (TC), triglycerides, low-density lipoprotein cholesterol, and high-density lipoprotein cholesterol. Liver enzymes and lipid parameters were measured in the fasting state upon admission or on the morning of the second day after admission.

### Definition of outcome indicators

This study followed up participants from the time of admission, with the follow-up endpoint being the occurrence of outcome events or the 30th day after admission, whichever occurred first. The primary outcome event was all-cause mortality in ADHF patients within 30 days of hospitalization.

### Statistical analysis

Participants were divided into three groups (low, moderate, and high) based on the tertiles of PWR. Normally distributed continuous data were expressed as mean (standard deviation), while non-normally distributed continuous data were expressed as median (interquartile range); categorical data were presented as frequency (percentage). Differences between groups were compared using Kruskal–Wallis *H*-test, one-way analysis of variance, or chi-square test depending on the data type.

First, Kaplan–Meier curves were plotted to assess the survival of ADHF patients in different PWR groups. Subsequently, several adjusted Cox regression models were constructed to evaluate the association between PWR and 30-day mortality in ADHF patients, with variance inflation factors less than 5 for covariates: Model 1 adjusted for gender, age, and comorbidities; Model 2 further adjusted for NYHA classification, LVEF, systolic BP, diastolic BP, and NT-proBNP based on Model 1; Model 3 adjusted for all non-collinear covariates. In addition, restricted cubic spline (RCS) regression was used to explore the dose-response relationship between PWR and 30-day mortality in ADHF patients. If a nonlinear association was detected, a recursive algorithm was applied to determine potential threshold points; Subsequently, a Cox regression model was created for each side of the threshold point, followed by a log-likelihood ratio test to determine whether a significant change occurred in the correlation between PWR and short-term mortality risk in ADHF patients before and after the threshold point.

We also conducted stratified analyses based on age, gender, NYHA classification, LVEF, and comorbidities such as hypertension, diabetes, cerebral infarction, and CHD, and analyzed potential interactions between these stratified variables and PWR using likelihood ratio tests. Finally, receiver operating characteristic curves were plotted to evaluate and compare the predictive value of PWR with the established ADHF biomarker NT-proBNP for all-cause mortality within 30 days of admission in ADHF patients, and the area under the curve (AUC) was calculated. Additionally, we evaluated the potential improvement in predictive performance by incorporating PWR into the NT-proBNP model, where AUC values were compared using the Delong test (37). All analyses in this study were performed using R language version 4.2.1 and Empower(R) version 2.20 statistical software, with a significance level set at *P* < 0.05 (two-sided).

## Results

### Study cohort

This study included 1,453 participants, of whom 837 (57.6%) were male and 616 (42.4%) were female. During the 30-day observation period, 53 (3.65%) participants experienced mortality events.

Table 1 presents the baseline characteristics of ADHF patients at admission grouped according to the tertiles of PWR. Compared to participants with moderate to high PWR levels, those in the low PWR group exhibited significantly higher levels of age, WBC, hemoglobin, ALT, AST, creatinine, and NT-proBNP, while lower levels of PLT, ALB, TC, high-density lipoprotein cholesterol, and low-density lipoprotein cholesterol. Additionally, a higher proportion of male participants, those with diabetes, and those classified as NYHA grade IV were observed in the low PWR group.

**Table 1:** Summary of baseline characteristics of the study population according to PWR tertiles group.

	PWR tertiles			*P*-value
	Low (2.30–22.25)	Moderate (22.31–30.70)	High (30.71–92.08)	
No. of subjects	484	484	485	
Gender				
Female	172 (35.54%)	204 (42.15%)	240 (49.48%)	<0.001
Male	312 (64.46%)	280 (57.85%)	245 (50.52%)	
Age (years)	72.00 (64.00–80.00)	69.00 (58.00–78.00)	68.00 (57.00–78.00)	<0.001
Comorbidities				
Hypertension (*n*, %)	211 (43.60%)	204 (42.15%)	185 (38.14%)	0.203
Diabetes (*n*, %)	143 (29.55%)	122 (25.21%)	105 (21.65%)	0.018
Cerebral infarction (*n*, %)	87 (17.98%)	76 (15.70%)	68 (14.02%)	0.240
CHD (*n*, %)	141 (29.13%)	148 (30.58%)	161 (33.20%)	0.382
NYHA classification (*n*, %)				
III	297 (61.36%)	351 (72.52%)	356 (73.40%)	<0.001
IV	187 (38.64%)	133 (27.48%)	129 (26.60%)	
SBP (mmHg)	128.42 (26.41)	126.77 (23.78)	129.08 (23.54)	0.323
DBP (mmHg)	76.07 (16.88)	75.70 (15.85)	75.58 (14.71)	0.878
LVEF (%)	48.00 (39.00–56.00)	48.00 (36.00–57.00)	48.00 (39.00–56.00)	0.658
WBC (×10^9^/L)	7.82 (6.10–10.81)	6.00 (4.90–7.40)	5.40 (4.40–6.40)	<0.001
RBC (×10^12^/L)	4.11 (0.80)	4.09 (0.76)	4.05 (0.79)	0.460
HGB (g/L)	124.50 (23.41)	124.00 (21.81)	119.77 (23.14)	0.002
PLT (×10^9^/L)	129.00 (93.00–164.00)	157.50 (130.00–196.00)	209.00 (172.00–251.00)	<0.001
ALB (g/L)	34.48 (5.43)	35.72 (4.83)	35.43 (4.75)	<0.001
ALT (U/L)	26.00 (15.00–48.50)	21.00 (14.00–35.00)	19.00 (12.00–30.00)	<0.001
AST (U/L)	30.00 (21.00–50.50)	26.00 (20.00–37.00)	23.00 (18.75–32.00)	<0.001
Cr (umol/L)	98.00 (75.00–149.00)	88.00 (67.50–113.00)	80.00 (61.00–109.00)	<0.001
TG (mmol/L)	1.10 (0.87–1.55)	1.17 (0.91–1.56)	1.18 (0.88–1.58)	0.381
TC (mmol/L)	3.63 (0.94)	3.79 (0.98)	4.01 (1.18)	<0.001
HDL-C (mmol/L)	0.96 (0.77–1.15)	0.98 (0.82–1.16)	1.02 (0.85–1.22)	0.011
LDL-C (mmol/L)	2.15 (1.65–2.70)	2.22 (1.81–2.86)	2.42 (1.85–3.03)	<0.001
NT-proBNP (pmol/L)	3,868.50 (2,452.75–5,844.75)	3,616.00 (2,044.00–5,931.00)	3,452.00 (1,893.00–5,473.00)	0.004
30-day mortality	33 (6.82%)	15 (3.10%)	5 (1.03%)	<0.001

CHD, coronary heart disease; NYHA, New York Heart Association; LVEF, left ventricular ejection fraction; SBP, systolic blood pressure; DBP, diastolic blood pressure; TG, triglyceride; TC, total cholesterol; HDL-C, high-density lipoprotein cholesterol; LDL-C, low-density lipid cholesterol; Cr, creatinine; WBC, white blood cell count; RBC, red blood cell count; HGB, hemoglobin; PLT, platelet count; ALT, alanine aminotransferase; AST, aspartate aminotransferase; ALB, albumin; NT-proBNP, N-Terminal Pro-Brain Natriuretic Peptide; PWR, platelet-to-white blood cell ratio.

**Figure 2:**
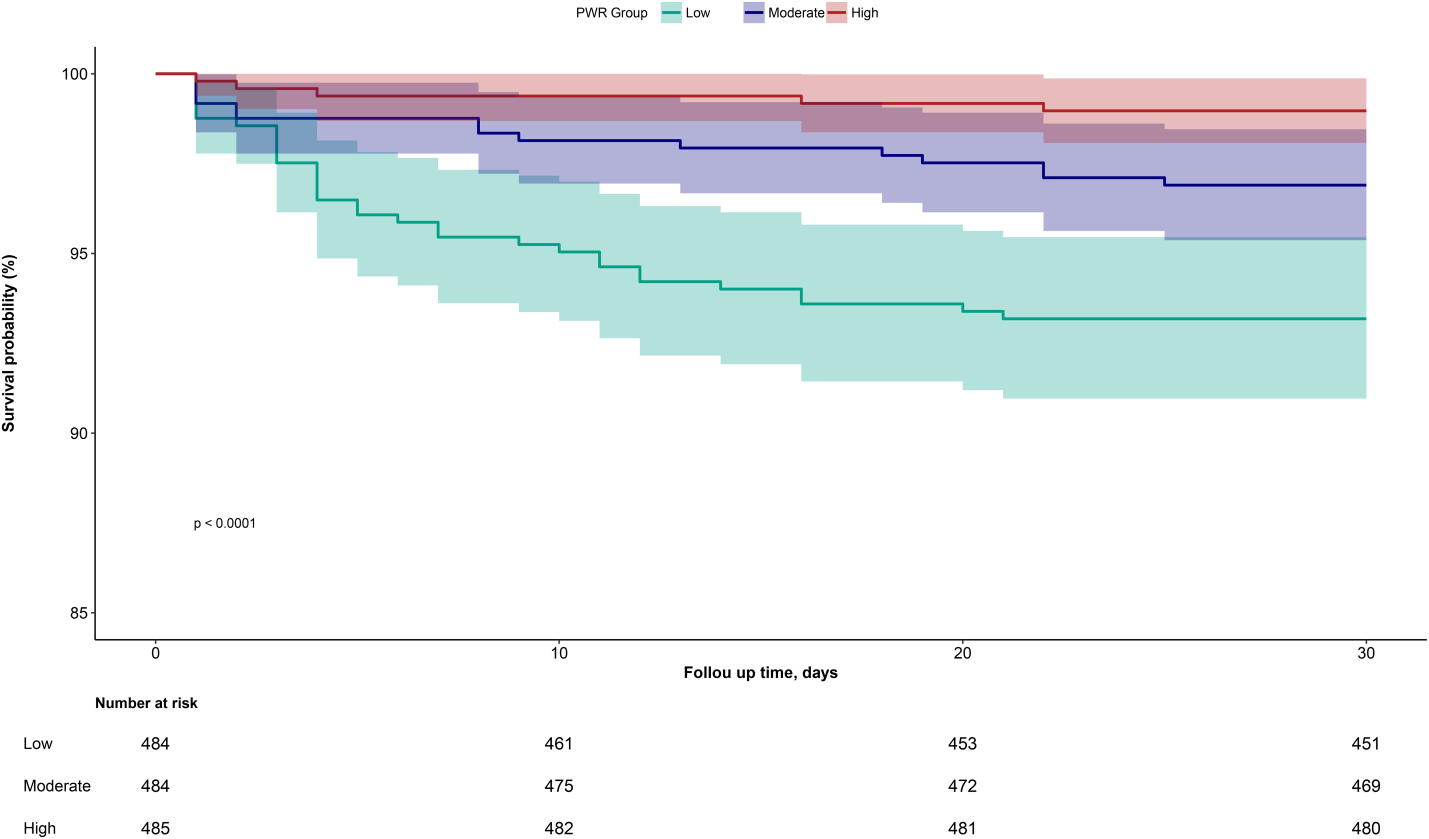
Cumulative survival rate curves of ADHF patients in PWR group.

Figure 2 illustrates the 30-day cumulative survival curves for ADHF patients in the low, moderate, and high PWR groups. The results indicated that compared to ADHF patients with moderate to high PWR levels, those with low PWR had a significantly higher mortality rate within 30 days (log-rank *P* < 0.05).

### Association between PWR and 30-day mortality in ADHF patients

We included PWR as both a continuous and categorical variable in four sequentially adjusted Cox regression models to explore its association with 30-day mortality in ADHF patients. Prior to establishing the multivariable Cox regression models, collinearity analysis was performed, with covariates having variance inflation factor values greater than 5 (ALT, TC) considered to have high collinearity and thus not included in subsequent models (Supplementary Table S1). When PWR was treated as a continuous variable, a negative correlation was observed between PWR and 30-day mortality after adjusting for all non-collinear covariates (Crude model HR: 0.90; Model 1 HR: 0.91; Model 2 HR: 0.93; Model 3 HR: 0.93). When PWR was treated as a categorical variable, compared to the low PWR group, the higher PWR groups exhibited stronger negative correlations with 30-day mortality risk of participants (Table 2). Specifically, the results of Model 3 showed that the 30-day mortality risk for ADHF patients in the high PWR group decreased by 76% compared to the low PWR group (HR 0.24, 95% CI: 0.06, 0.86). In summary, low PWR emerged as an independent risk factor for all-cause mortality during hospitalization in ADHF patients.

**Table 2:** Multivariable Cox regression analysis of the association between PWR and 30-day mortality in patients with ADHF.

	Hazard ratios (95% confidence interval)			
	Crude model	Model 1	Model 2	Model 3
PWR (continuous variable)	0.90 (0.87, 0.93)	0.91 (0.88, 0.94)	0.93 (0.90, 0.96)	0.93 (0.89, 0.97)
PWR (tertiles)				
T1 (Low)	Ref	Ref	Ref	Ref
T2 (Moderate)	0.45 (0.24, 0.82)	0.51 (0.28, 0.95)	0.66 (0.34, 1.28)	0.80 (0.37, 1.75)
T3 (High)	0.15 (0.06, 0.38)	0.18 (0.07, 0.45)	0.29 (0.11, 0.78)	0.24 (0.06, 0.86)
*P*-trend	<0.0001	<0.0001	0.0099	0.0319

Model 1 adjusted for gender, age, hypertension, diabetes, cerebral infarction and CHD.

Model 2 adjusted for model 1+ NYHA classification, LVEF, SBP, DBP, NT-proBNP.

Model 3 adjust for Model 2+ RBC, Hb, ALB, AST, TG, HDL-C, LDL-C and Cr.

### Dose-response relationship between PWR and 30-day mortality risk in ADHF patients

**Figure 3:**
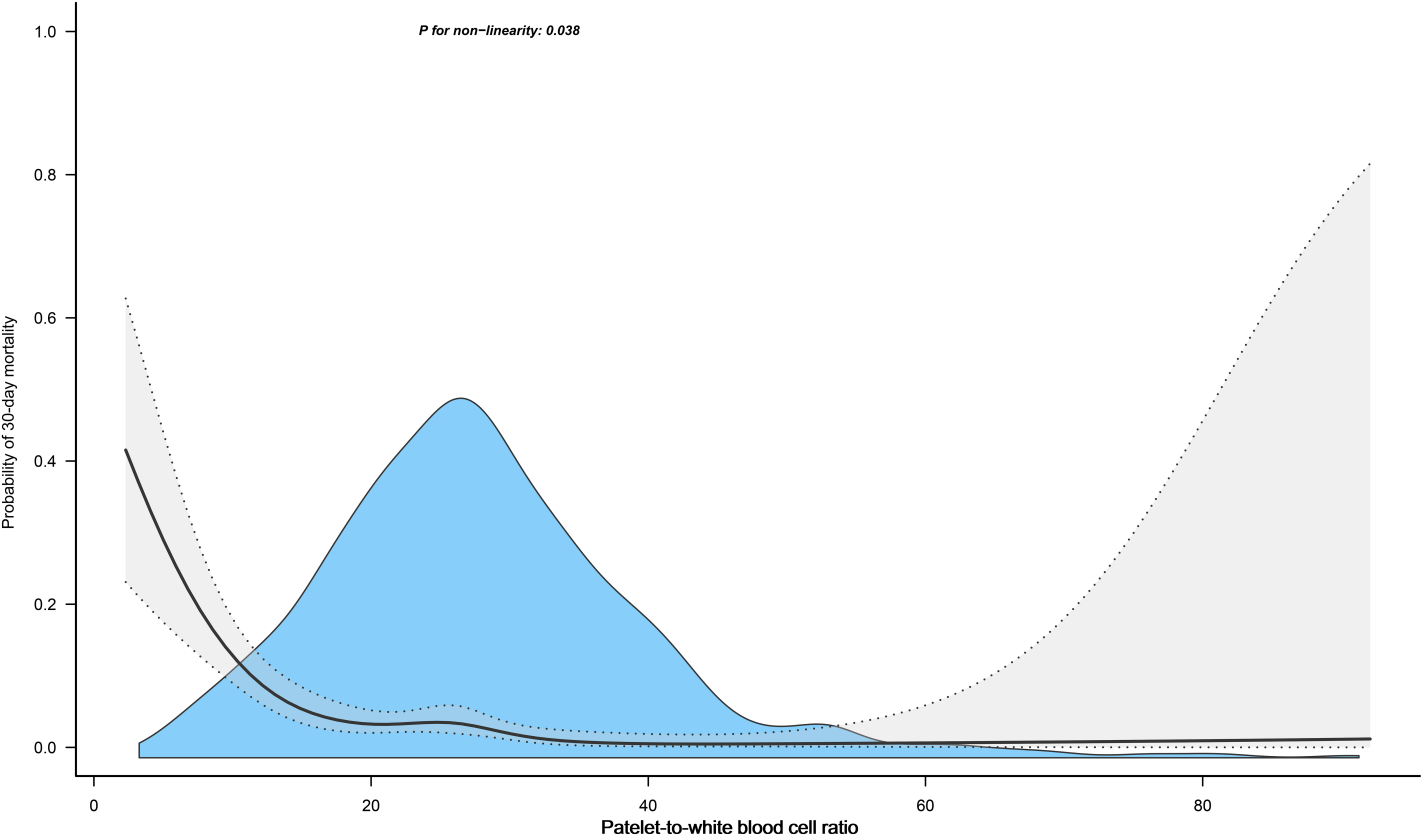
Fitting the dose-response relationship between PWR and 30-Day All-cause mortality in ADHF patients with 4 knots restricted cubic spline. Adjusted for gender, age, hypertension, diabetes, cerebral infarction, CHD, NYHA classification, LVEF, SBP, DBP, NT-proBNP, RBC, Hb, ALB, AST, TG, HDL-C, LDL-C and Cr.

We further assessed the dose-response relationship between PWR and the risk of 30-day mortality in ADHF patients using RCS regression models. As depicted in Figure 3, there was a nonlinear L-shaped association between PWR and 30-day mortality, with a potential threshold effect point (between 15 and 20). When PWR was below this threshold point, the risk of all-cause mortality in ADHF patients significantly decreased with increasing PWR, whereas the curve flattened when PWR exceeded this threshold point. We further employed segmented Cox regression analysis to calculate the optimal inflection point on the dose-response relationship curve between PWR and all-cause mortality risk, revealing the optimal inflection point to occur at a PWR of 15.88 (Table 3), where a decrease in PWR below this inflection point was significantly associated with increased risk of all-cause mortality.

**Table 3:** The result of the two-piecewise Cox regression model.

	30-day mortality (HR, 95% CI)
Fitting model by two-piecewise Cox regression	
Inflection points of PWR	15.88
<15.88	0.84 (0.76, 0.93) 0.0011
>15.88	0.97 (0.92, 1.03) 0.2931
*P* for log-likelihood ratio test	0.046

HR, hazard ratios; CI, confidence interval.

Adjusted for gender, age, hypertension, diabetes, cerebral infarction, CHD, NYHA classification, LVEF, SBP, DBP, NT-proBNP, RBC, Hb, ALB, AST, TG, HDL-C, LDL-C and Cr.

### ROC analysis

We also evaluated and compared the predictive value of PWR and NT-proBNP for predicting mortality events within 30 days of admission in ADHF patients using receiver operating characteristic curves (Table 4). The study findings indicated that PWR exhibited a higher predictive value for 30-day mortality events in ADHF patients compared to NT-proBNP (AUC: NT-proBNP 0.69, PWR 0.76; Delong *P* < 0.05). Notably, when PWR was added to the NT-proBNP model for predicting 30-day mortality, we observed a significant enhancement in the model's predictive capability, with the AUC increasing from 0.69 to 0.80 (Delong test *P* < 0.01). These results underscored the importance of further evaluating the novel inflammatory marker PWR in predicting mortality risk among ADHF patients.

**Table 4:** ROC curves analyzing the predictive ability of NT-proBNP, PWR for 30-day mortality and the improvement of 30-day mortality prediction by adding PWR on top of NT-proBNP.

	AUC	95% CI low	95% CI upp	Best threshold	Specificity	Sensitivity
NT-proBNP*^,^**	0.69	0.61	0.77	5,102.50	0.69	0.68
PWR	0.76	0.69	0.82	14.92	0.90	0.49
NT-ProBNP + PWR	0.80	0.73	0.86	−3.04	0.80	0.68

AUC, area under the curve; other abbreviations as in Table 1.

* *P* < 0.01, compare with PWR (Delong test).

** *P* < 0.01, compare with NT-ProBNP+PWR (Delong test).

### Subgroup analysis

To explore whether the association between PWR and the risk of all-cause mortality differed among ADHF patients with different characteristics, we conducted subgroup analyses based on age, gender, NYHA classification, LVEF, and the presence of comorbidities such as hypertension, diabetes, cerebral infarction, and CHD. Significant differences of the association were observed only in the LVEF subgroup: compared to patients with HF with reduced ejection fraction (LVEF <40%) and those with HF with mid-range ejection fraction (LVEF 40%–49%), patients with HF with preserved ejection fraction (HFpEF: LVEF ≥50%) had a stronger negative correlation between PWR and 30-day mortality (HR: 0.99 vs. 0.98 vs. 0.87, *P* = 0.0067) (Table 5).

**Table 5:** Stratified analysis showed the relationship between PWR and 30-day mortality in patients with ADHF in different age, gender, NYHA class, LVEF and whether combined with hypertension/diabetes/cerebral infarction/CHD.

Subgroup	Adjusted HR (95% CI)	*P* for interaction
Age (years)		
20–70	0.89 (0.82, 0.97)	0.1872
71–100	0.95 (0.90, 0.99)	
Gender		
Male	0.93 (0.89, 0.98)	0.7645
Female	0.92 (0.85, 0.99)	
NYHA class		
III	0.91 (0.85, 0.98)	0.4321
IV	0.94 (0.89, 0.99)	
LVEF		
HFrEF (LVEF <40%)	0.99 (0.93, 1.05)	0.0067
HFmrEF (LVEF 40%–49%)	0.98 (0.91, 1.06)	
HFpEF (LVEF ≥50%)	0.87 (0.81, 0.93)	
Hypertension		
Yes	0.92 (0.86, 0.98)	0.5646
No	0.94 (0.89, 0.99)	
Diabetes		
Yes	0.92 (0.86, 1.00)	0.8519
No	0.93 (0.89, 0.98)	
Cerebral infarction		
Yes	0.94 (0.88, 1.00)	0.7552
No	0.93 (0.88, 0.97)	
CHD		
Yes	0.94 (0.89, 1.00)	0.5354
No	0.92 (0.87, 0.97)	

HFrEF, heart failure with reduced ejection fraction; HFmrEF, heart failure with mid-range ejection fraction; HFpEF, heart failure with preserved ejection fraction; other abbreviations as in Table 1.

Models adjusted for the same covariates as in model 3 (Table 2), except for the stratification variable.

## Discussion

In this retrospective cohort study, we analyzed clinical data from 1,453 ADHF patients in Jiangxi, China, and revealed, for the first time, the association between PWR and the risk of all-cause mortality within 30 days of admission among Chinese ADHF patients. The results indicated that after controlling for confounding factors, PWR was negatively correlated with the risk of 30-day all-cause mortality, particularly when PWR was <15.88, where the risk of death in ADHF patients was significantly decreased with increasing PWR.

Hospitalization of ADHF patients is closely associated with high mortality and readmission rates (9–11, 38). Early identification of important risk factors during hospitalization for ADHF patients is crucial for preventing or reducing mortality rates. Previous studies have shown that systemic organ congestion is a significant factor contributing to adverse events in ADHF patients (39, 40). With further understanding of the disease, research has revealed that inflammatory reactions also contribute to the exacerbation of ADHF (13, 41). Inflammatory responses mediate the occurrence and development of diseases such as atherosclerosis (42), hypertension (43), and myocardial infarction (44), playing a crucial role in cardiovascular diseases. ADHF represents a common manifestation of advanced stages of various cardiovascular diseases (45), and numerous studies have demonstrated that inflammatory biomarkers can serve as indicators for evaluating the prognosis of ADHF patients. For example, Zhu X et al. constructed a new inflammation prognosis score system by incorporating different inflammatory indicators such as C-reactive protein, red cell distribution width, and neutrophil-to-lymphocyte ratio, which significantly improved the predictive ability of the model and can be used as a practical tool for individualized risk stratification of ADHF patients (46). Other studies have indicated that C-reactive protein and interleukin-6 are predictive factors for all-cause mortality in AHF patients (47, 48). Additionally, Ye GL et al. found in a cohort study of 443 AHF patients from northern China that the inflammatory marker PLT-to-lymphocyte ratio is independently correlated with all-cause mortality in ADHF patients; the higher the PLT-to-lymphocyte ratio, the greater the risk of mortality in ADHF patients (HR 2.437; 95% CI 1.302, 3.653) (49).

PWR is a novel biomarker reflecting systemic inflammation, with a decrease in this parameter indicating disruption of immune balance (50). As indispensable factors in the immune system, both PLT and WBC typically exhibit a sharp increase during acute infections (51, 52). However, in critically ill patients, PLT often remain at low levels, which may increase the risk of bleeding and mortality (53, 54). A cohort study from Japan found that a decrease in PLT level increases the risk of mortality and readmission in ADHF patients (55). Furthermore, compared to survivors, non-survivors of ADHF patients often have higher baseline levels of WBC upon admission, although this is not significantly correlated with the risk of death, which may be due to differences in the study population (56). Therefore, for acute HF populations, both decreased PLT and increased WBC levels increase the risk of mortality during hospitalization. As a composite index combining PLT and WBC, the PWR provides a more comprehensive assessment of disease severity. In the current study, we further identified PWR as an important biomarker for assessing short-term prognosis in patients with ADHF and significantly superior to the traditional HF marker NT-proBNP. Integrating previous studies (23–36) with current research findings, PWR appears to be a promising new marker in the field of critical illness. We suggest that healthcare workers in the emergency room as well as the intensive care unit need to focus on the PWR of their patients.

Systemic inflammatory responses are widely present in various human diseases, providing important protection against adverse factors (57). After establishing the association between PWR and short-term mortality prognosis in patients with ADHF, identifying critical PWR thresholds in different clinical scenarios may be important for clinical practice. In terms of chronic disease assessment, analyses of previous studies have shown an L-shaped association between PWR and diabetes, chronic kidney disease, and stroke similar to the current study, with an associated cutoff point of approximately 30 (17, 18, 22). In addition, in the assessment of critical illnesses, some studies have also calculated critical values for the prediction of poor prognosis by ROC analysis: A study from China analyzed the association between PWR and mortality in patients with subarachnoid hemorrhage and found that lower PWR was associated with increased risk of postoperative pneumonia and death (optimal cutoff point was 15.69) (25). Another study found that the risk of death significantly increased in patients with acute novel coronavirus infection when PWR was below 20.34 (26). A study following 269 pancreatic cancer patients found that a decrease in PWR provided a suitable environment for tumor cell growth, particularly when PWR was <6, where the risk of death was significantly elevated (29). Additionally, a study from Korea showed that PWR had evaluative significance for short-term adverse outcomes in patients with acute decompensated cirrhosis, with a threshold point of 12.1 (27). In our current analysis, through RCS analysis, we found that PWR has an L-shaped association with 30-day mortality risk in ADHF patients, with the PWR-related risk of death cutoff point being 15.88; in addition, by ROC analysis we also calculated the optimal threshold for predicting 30-day mortality in patients with ADHF to be 14.92; these findings are similar to several previous reports (25–27). Overall, PWR has a wide range of applications and has good potential for promotion. Relatively speaking, in the context of chronic disease risk assessment, the cutoff point for PWR is relatively lenient, with a recommended PWR value maintained below 30. However, for predicting adverse prognoses in acute and critical illnesses, a stricter criterion for the PWR cutoff point is required, with a recommended PWR value kept below 15.

In our subgroup analysis, we also identified a particular finding: among patients with HFpEF in ADHF, there existed a stronger negative correlation between PWR and 30-day mortality compared to those with reduced or intermediate ejection fractions. This suggests that, at equivalent PWR levels, HFpEF patients have a greater protective effect. Similarly, at equivalent PWR levels, ADHF patients with reduced and mid-range ejection fractions face a higher risk of mortality, indicating that the deterioration of cardiac systolic function further exacerbates the mortality risk associated with low PWR. It is well recognized that in the setting of inadequate effective circulating blood volume, myocardial cells mount complex inflammatory responses to danger signals, primarily involving: (1) the expression of pro-inflammatory and anti-inflammatory cytokines such as IL-6 and IL-10, initiating and modulating local inflammatory reactions (58); (2) the expression of chemotactic factors like KC and MIP-2, recruiting and activating appropriate subsets of inflammatory cells for response and repair (59, 60); and (3) the expression of cell surface adhesion molecules, particularly ICAM-1, facilitating interactions between inflammatory cells and the extracellular matrix as well as signaling cascades from the extracellular milieu to the intracellular environment (61–63). In the scenario of decreased cardiac contractility, these cytokines, chemotactic factors, subsequently recruited leukocytes, and cell surface adhesion molecules may instigate a cascade of inflammatory reactions, influencing the cardiac repair processes, ultimately leading to mortality (58–63). Considering the stronger negative correlation between PWR and mortality in HFpEF patients, which provides more protective information by comparison, close monitoring of inflammatory biomarkers such as PWR changes may be deemed more imperative, particularly for patients with HFpEF.

The current research findings have significant reference value for patients with ADHF. ADHF is known to have severe symptoms, rapid onset of seizures, and poor short-term prognosis, making it one of the most difficult inpatient diseases to properly manage in the clinic (3–8). In the current study, we tested the association of a facile novel inflammatory marker, PWR, with the 30-day prognosis of death in patients with AHDF and determined that PWR has an important application in the short-term prognostic assessment of patients with ADHF and is significantly superior to the traditional HF marker NT-proBNP. It should be noted that PWR is simple to obtain and requires only routine blood analysis (16), which is a very routine measurement in community clinics as well as in hospitals of different levels. Therefore, we believe that PWR can be a promising tool for risk stratification and prognostic assessment of patients with ADHF. Of course, we also hope that the results of the current study will be further validated in the future in other regions and races and will inform the development of future HF management guidelines.

## Advantages and limitations

### Advantages

(1) This study is the first to discover a negative correlation between PWR and all-cause mortality in ADHF patients within 30 days of hospital admission among the Chinese population. (2) PWR, composed of PLT and WBC, can be easily obtained through routine blood tests. (3) This study revealed an L-shaped correlation between PWR and 30-day mortality rate, identifying a PWR threshold of 15.88.

### Limitations

(1) The observational nature of the study limits further assessment of the impact of treatments targeting PWR on prognosis, necessitating further prospective research. (2) The study population mainly consists of individuals from various regions in Jiangxi, China, caution should be exercised when extrapolating the study findings to populations from other countries or provinces in China. (3) Despite extensive adjustment for covariates in the current analysis, some unmeasured factors may not have been included in the study. (4) Due to the lack of repeated measurement data for PWR, it is not possible to further explore the risk assessment value of PWR for short-term mortality in ADHF patients.

## Conclusion

This study demonstrates a negative correlation between the inflammatory marker PWR and short-term mortality in ADHF patients, and its predictive performance for short-term death events is significantly better than the traditional HF marker NT-proBNP. Based on the findings of this study, we recommended using PWR to assess the risk of mortality in ADHF patients during hospitalization, providing valuable guidance for clinical management.

## Data Availability

The raw data supporting the conclusions of this article will be made available by the authors, without undue reservation.
