# Supplementary material for: Assessment of platelet-to-white blood cell ratio on short-term mortality events in patients hospitalized with acute decompensated heart failure: evidence from a cohort study from Jiangxi, China
Source: Front Cardiovasc Med. 2025 Feb 7;12:1454933. doi: 10.3389/fcvm.2025.1454933 (PMC11842369; doi:10.3389/fcvm.2025.1454933)
Supplement: Supplementary file 1 [file Table1.docx]

Supplementary Table 1: Collinearity diagnostics steps.

|  | VIF | | |
| --- | --- | --- | --- |
|  | Step 1 | Step 2 | Step 3 |
| PWR | 4 | 4 | 4 |
| Gender | 1.2 | 1.2 | 1.2 |
| AGE | 1.4 | 1.4 | 1.4 |
| Hypertension | 1.3 | 1.3 | 1.3 |
| Diabetes | 1.1 | 1.1 | 1.1 |
| Stroke | 1.1 | 1.1 | 1.1 |
| CHD | 1.2 | 1.2 | 1.2 |
| NYHA classification | 1.1 | 1.1 | 1.1 |
| SBP | 1.8 | 1.8 | 1.8 |
| DBP | 1.5 | 1.5 | 1.5 |
| LVEF | 1.2 | 1.2 | 1.2 |
| WBC | 3.3 | 3.3 | 3.3 |
| RBC | 4.1 | 4.1 | 4.1 |
| HGB | 4.3 | 4.3 | 4.3 |
| PLT | 3.7 | 3.6 | 3.6 |
| ALB | 1.3 | 1.3 | 1.3 |
| ALT | 7.4 | 7.4 | NA |
| AST | 7.3 | 7.3 | 1.2 |
| Cr | 1.4 | 1.3 | 1.3 |
| TG | 2.1 | 1.1 | 1.1 |
| TC | 11.7 | NA | NA |
| HDL-C | 2.6 | 1.3 | 1.3 |
| LDL-C | 8.2 | 1.2 | 1.2 |
| NT-proBNP | 1.2 | 1.2 | 1.2 |

VIF: variance inflation factor; VIF = 1/(1-R^2^). Abbreviations as in Table ​1.

Note: The variables with VIF>5 will be regarded as collinear variables and cannot be included in the multiple regression model.
